# 630. SARS COV-2 genomic surveillance in Pakistan, an innovative approach

**DOI:** 10.1093/ofid/ofad500.696

**Published:** 2023-11-27

**Authors:** Saeed Ahmad, Fahmeeda Idrees

**Affiliations:** Health Services Academy, Islamabad, Pakistan, Islamabad, Islamabad, Pakistan; Health Services Academy, Islamabad, Pakistan, Islamabad, Islamabad, Pakistan

## Abstract

**Background:**

Novel strains of severe acute respiratory syndrome coronavirus 2 (SARS-CoV-2)harboring diverse gene mutations have periodically emerged and are spreading globally. Importantly, mutations within the spike gene's receptor-binding domain (RBD)are linked to SARS-CoV-2 transmissibility, disease severity, or decreased diagnostics vaccine efficacy. To identify SARS-CoV-2 variants rapidly,

**Methods:**

we used a single primer-based PCR method in conjunction with Sanger sequencing. We focused on a region of the spike gene's RBD and S2 domains that contained lineage-defining mutational combinations.

**Results:**

The genetic data obtained showed that delta and omicron subvariants were prevalent in the Khyber Pakhtunkhwa (KPK) region from January to February2022. Our survey found the highest prevalence of SARS-CoV-2 in the city of Peshawar (5.2%), followed by the districts of Mardan (1.8%), Karak (1.4%), Malakand(0.6%), and other districts.

**Conclusion:**

We recommend that SARS-CoV-2 be monitored continuously to detect single-nucleotide polymorphisms (SNPs) that are causally linked to the emergence of new lineages. In resource-constrained settings, our method provides a rapid and cost-effective detection assay for SARS-CoV-2.

**Disclosures:**

**All Authors**: No reported disclosures

